# Inhibition of 2A-mediated ‘cleavage’ of certain artificial polyproteins bearing *N*-terminal signal sequences

**DOI:** 10.1002/biot.200900134

**Published:** 2010-02

**Authors:** Pablo de Felipe, Garry A Luke, Jeremy D Brown, Martin D Ryan

**Affiliations:** 1 Centre for Biomolecular Sciences, North Haugh, University of St. AndrewsSt. Andrews, Scotland, UK; 2 RNA Biology Group and Institute for Cell and Molecular Biosciences, The Medical School, Newcastle UniversityNewcastle upon Tyne, UK

**Keywords:** 2A, Polyprotein, Skipping, Slipstream, Targeting

## Abstract

Where 2A oligopeptide sequences occur within ORFs, the formation of the glycyl-prolyl peptide bond at the C-terminus of (each) 2A does not occur. This property can be used to concatenate sequences encoding several proteins into a single ORF: each component of such an artificial polyprotein is generated as a discrete translation product. 2A and ‘2A-like’ sequences have become widely utilised in biotechnology and biomedicine. Individual proteins may also be co- and post-translationally targeted to a variety of sub-cellular sites. In the case of polyproteins bearing N-terminal signal sequences we observed, however, that the protein downstream of 2A (no signal) was translocated into the endoplasmic reticulum (ER). We interpreted these data as a form of ‘slipstream’ translocation: downstream proteins, without signals, were translocated through a translocon pore already formed by the signal sequence at the N-terminus of the polyprotein. Here we show this effect is, in fact, due to inhibition of the 2A reaction (formation of fusion protein) by the C-terminal region (immediately upstream of 2A) of some proteins when translocated into the ER. Solutions to this problem include the use of longer 2As (with a favourable upstream context) or modifying the order of proteins comprising polyproteins.

## 1 Introduction

2A and ‘2A-like’ oligopeptide (20–30aa) sequences (‘2As’) are widely used in biotechnology and biomedicine to co-express multiple different proteins within the same cell. Genes encoding proteins are linked *via* 2A sequences to form a single ORF. Translation of the single ORF results, however, in the biosynthesis of each protein as a discrete translation product [1–10]. The great utility here is that multiple proteins comprising heteromultimers, macromolecular assemblies, biochemical pathways, *etc.*, plus (selectable) marker proteins may be assembled into, and co-expressed from, a single transgene. Since 2As function in all eukaryotic systems tested to date, they have found utility in animal, plant and microbial biotechnologies (http://www.st-andrews.ac.uk/ryanlab/Index.htm and reviewed in [11, 12]). Notably, this system has recently been used in adoptive cell therapies (ACT) [13–18], genetic engineering of human stem cells [19–21] and the co-expression of transcription factors in the induction of pluripotent stem cells [22–27].

2As are neither proteolytic elements nor substrates for cellular proteinases, but mediate a newly discovered form of translational recoding event referred-to variously as ribosome ‘skipping’,‘stop-go’ and ‘stop-carry on’ translation [5, 8, 9]. When a ribosome encounters 2A within an ORF it ‘skips’ the synthesis of the glycyl-prolyl peptide bond at the C-terminus of 2A. The nascent protein is released from the ribosome by eukaryotic release (termination) factors 1 and 3 (eRF1, eRF3), 2A forming its C-terminus [9]. The ribosome then resumes translation of the downstream sequences. As one test of our intra-ribosomal, co-translational, model for the mechanism of the 2A reaction, we assembled complementary DNA (cDNA) encoding a synthetic polyprotein comprising a protein targeted to the exocytic pathway (prepro-αfactor; αF), 2A, and green fluorescent protein (GFP). Expression in yeast showed that the translation product upstream of 2A was, indeed, targeted to the endoplas-mic reticulum (ER) whilst the downstream product localised to the cytosol. The 2A reaction occurred even though the nascent protein was ‘shielded’ from cytosolic proteinases by the establishment of a ribosome:translocon complex [7].

To extend the utility of 2A in the co-expression of proteins targeted to different sub-cellular compartments, we constructed plasmids encoding a panel of polyproteins similar to those used in our yeast analyses. Our ‘basic’ polyprotein construct comprised enhanced yellow fluorescent protein (EYFP), 2A, enhanced cyan fluorescent protein (ECFP), a second 2A and puromycin resistance (PAC; plasmid pPDF20). All proteins were co-expressed and located to the cytoplasm [28]. This construct was modified by the insertion of a GalT type II signal-anchor sequence (β-1,4 galactosyltransferase, GT) onto the N-terminus, to encode a [GT-EYFP-2A-ECFP-2A-PAC] polyprotein (plasmid pPDF18). When analysed using translation systems *in vitro* the expected translation profile was observed: high-level ‘cleavage’ (>90%) at both of the 2As, producing the major translation products [GT-EYFP-2A], [ECFP-2A] and PAC. When this construct was used to transfect HeLa cells, however, whilst the fluorescence signal from EYFP (correctly) localised in the Golgi, the signal from ECFP localised to the ER [28]. The latter was unexpected since [ECFP-2A] did not bear a signal sequence. Furthermore, this result was at variance with our findings in yeast, where the protein downstream of 2A localised to the cytosol and was also at variance with reports in the literature where secreted heterodimers were co-expressed using 2A (discussed below).

Our conclusion from the data derived from the fluorescent proteins bearing N-terminal co-translational signal sequences (types I and II) was that (i) translation of the first protein (bearing a signal sequence) lead to the formation of the ribosome:translocon (Sec61) complex, (ii) the N-terminal protein was translocated into the ER and (iii) that the protein downstream of 2A (no signal sequence) simply ‘slipstreamed’ through the pore of the translocon complex already formed [28].

Here we present data that shows this interpretation to be incorrect. We show that an interaction between the C-terminal region of *certain* nascent peptides and the translocon complex can affect the structure of the C-terminus of 2A within the pep-tidyl-transferase centre of the ribosome. This leads to inhibition of the 2A reaction, greatly increasing peptide bond formation and the production of ‘un-cleaved’ fusion proteins. The fluorescence patterns we observed were primarily due, therefore, to the localisation and, to some extent, the fluorescent properties of these uncleaved forms.

## 2 Materials and methods

### 2.1 Cloning

Plasmids pPDF18, pPDF19, pPDF20 and pPDF67 were as described previously [28]. Plasmids encoding the fluorescent proteins EYFP and ECFP and the GT signal were purchased from Clontech. The intermediate plasmid pPDF15 [GT-EYFP-2A-PAC] was derived from pPDF9 [GFP-2A-PAC] [28], the GFP removed with *Nhe*I/*Xba*I and replaced with GT-ECFP amplified from pECFP-Golgi (Clontech) using the primers PDFK (5′-CAGATCCGCTAGC ATGAGGCTTCGGGAG-3′) and 3′egfp/*Xba*I (5′-GGCCTCTAGAC TTGTACAGCTCGTCCAT-3′). The ECFP was then removed by restriction with *Bam*HI/*Xba*I and replaced with the PCR product EYFP (restricted with *Bgl*II and *Xba*I) from pEYFP-C1 (Clontech) using the primers PDFN (5′-GGCAGCAGATCTGGCTAGCATGAGGGTGAGC AAGGGCGAG-3′) and 3′egfp/*Xba*I. To obtain plasmid pPDF45ΔB the 2A-GT-ECFP ‘cassette’ was first removed from pPDF19 by *Xba*I restriction then ligated into pTG394pac [29] doubly restricted with *Xba*I and *Spe*I. The 3′pac-2A-GT-ECFP portion of the insert was then removed by *Bgl*II restriction, blunt-ended (Klenow fill-in) and restricted with *Sac*II. The restriction fragment was then ligated into pPDF15 restricted with *Xho*I, blunt-ended (Klenow fill-in) and *Sac*II to produce plasmidpPDF41 (encodes [GT-EYFP-2A-PAC-2A-GT-ECFP]). Finally, restrictions with *Apa*I followed by *Bam*HI removed the pac-2A and GT sequences, respectively.

Yeast signal sequences were amplified as αF-2A and D_N_αF-2A fragments from plasmids ppαF-2A-GFP and D_N_αF-2A-GFP [7] using the primers JN1 (5′-CCAGCTAGCGAATTCGTCGACCTCGAG-3′) and JN2 (5′-AAAACCATGGGCCCAGGGTTGG-3′) [7]. The PCR products were restricted with *Nhe*I/*Apa*I and used to replace the GT-EYFP-2A of pPDF45, similarly restricted. The yeast signals were removed from these plasmids with *Nhe*I/*Xba*I and used to replace the GT signal-anchor sequence excised from pPDF45ΔB with *Nhe*I/*Nhe*I, to give pPDF90 [D_N_αF-EYFP-2A-ECFP] and pPDF91 [αF-EYFP-2A-ECFP], respectively. pPDF45ΔB was restricted with *Nhe*I and *Xba*I to remove the EYFP and form plasmid pPDF87. To obtain pGL1, pPDF45ΔB was doubly restricted with *Spe*I and *Xba*I to remove the 3′ end of the cytomegalovirus (CMV) promoter, the T7 promoter, the GT signal and EYFP. This sequence was replaced with a similar fragment (with the shorter 5′ end of EYFP), produced by PCR amplification of pPDF15 using primers PDFY (5′-GACAATTGCATGAAGAATC-3′) and PDFX.30 (5′-CTCGCCTCTAGAGCTGAAC TTGTGGCCGTTTAC-3′), similarly restricted. pPDF45ΔB was restricted with *BspE*I/*Xba*I to remove EYFP, and replaced with a 3′ deleted version of the gene generated by PCR using plasmid pPDF15 as the template and oligonucleotides T7 (5′-TAATACGACTCACTATAGGG-3′) and PDFX.180 (5′-GTAGTGTCTAGAGAGCTGCACGCTGCCGT CCTC-3′) to form pAN1.9. Fortuitously, this reverse primer also hybridised 120 nucleotides downstream to give us an additional in-frame deleted version of the EYFP gene (pAN1.1)

Deletions in the EYFP gene in plasmid pPDF67 were introduced by replacing the *Bgl*II/*BsrG*I restriction fragment (containing CMV/T7 promoters, GT signal and EYFP) with equivalent fragments (having 3′ deleted versions of EYFP) from pGL1 to obtain pPDF107 and from pPDF87 to obtain pGL3. Plasmid pGL11 was obtained from pPDF67 by deleting the full EYFP gene, plus 2A, with *BstX*I/*BamH*I and the insertion of a small PCR fragment of EYFP, plus 2A (amplified using primers PDFX.30b; 5′-TGGAGTCCAAGGATCTGGCTCACAACGTCTATATCATGGC-3′ and SEQ4; 5′-ACCATGGTGGCGACCGGTGG AT-3′), similarly restricted, recreating the EYFP-2A junction present in pPDF116. Plasmid pPDF67 was restricted with *BstX*I and *SgrA*I to delete EYFP, which was replaced with the equivalent fragment from pAN1.9, or pAN1.1, to obtain pPDF115 or pPDF116, respectively. Progressively smaller 3′ deletions in EYFP were introduced in pPDF67 restricted with *BstX*I

and *SgrA*I. The deleted EYFP sequences were generated from PCR of pPDF15 by using the primers T7 and PDFX.200 (5′-CAGGGCCACCGGTGCGCA TGCTCTAGAGTTGTCGGGCAGCAGCACGGGG CCG-3′) producing pPDF117 or using primers T7 and PDFX.220 (5′-GCGGCCACCGGTGCGCATGCTCTAGACATGTGATCGCGCTTCTCGTTGGG-3′), to produce pPDF118.

### 2.2 *In vitro* translation

Coupled transcription/translation assays were performed using rabbit reticulocyte lysates (Promega) as described [7].

### 2.3 Cell culture, transfection and imaging

HeLa cells were grown in DMEM supplemented with 10% foetal calf serum. HeLa cells were seeded, transfected with Genejuice (Novagen), subsequently fixed (24 h), and imaged using DeltaVision microscope system (Applied Precision) as described [28].

### 2.4 Immunofluorescence

Transfected cells were fixed and then incubated with primary antibodies, either; (i) rabbit polyclon-al anti-2A (kind gift of Dr D. Vignali) or (ii) mouse monoclonal anti-V5 (kind gift of Prof. R. Randall). Texas red goat anti-rabbit and Texas red goat anti-mouse (Molecular Probes) were used as secondary antibodies.

### 2.5 Western blotting

HeLa cells were transfected and lysates collected 48 h later. Samples were run in 10 or 12.5% SDS–PAGE gels, transferred to Immobilon-P membranes (Millipore). Membranes were probed with primary antibodies, either (i) mouse monoclonal anti-GFP (Roche), (ii) anti-V5 or (iii) anti-2A antibodies. Secondary antibodies used were ECL anti-mouse IgG-Peroxidase from sheep (GE Healthcare) and anti-rabbit IgG-Peroxidase from goat (Sigma). Membranes were developed using ECL Plus Western Blotting Detection System (GE Healthcare).

## 3 Results

### 3.1 Sub-cellular localisation

**Figure 1:**
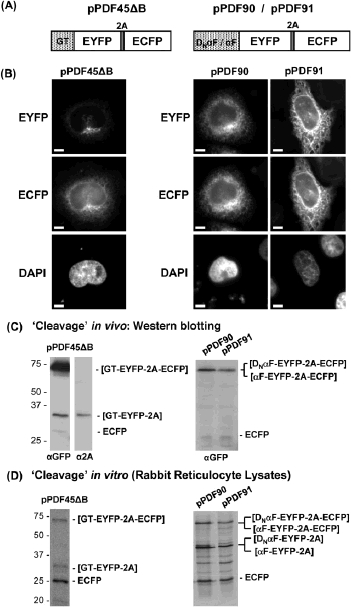
Cleavage activities and sub-cellular localisation of products derived from 2A-containing polyproteins. N-terminal signal sequences (stippled, boxed, areas) were derived from either GT, yeast α factor (αF) or the yeast Dap2p signal sequence (D_N_αF). Single ORFs were assembled to comprise; (i) an N-terminal signal sequence (GT – pPDF45ΔB; D_N_αF – pPDF90;αF – pPDF91), (ii) a fluorescent protein marker (EYFP; open boxed area), (iii) 2A (shaded, boxed, area) and (iv) a second fluorescent protein marker (ECFP; open boxed area) (panel A). In all cases protein expression was driven by a CMV promoter in the plasmid backbone. HeLa cells were transfected with plasmid constructs and images taken 24 h later (scale bar = 10 μm). The GT N-terminal signal-anchor directed EYFP to the Golgi and ECFP to the ER. This was also the case for proteins containing N-terminal yeast signal sequences (panel B). Cell lysates were prepared 24 h post-transfection, proteins separated by SDS–PAGE and subsequently analysed by Western blotting using antibody probes as indicated (panel C). Rabbit reticulocyte coupled transcription/ translation *in vitro* systems were programmed with plasmid cDNA constructs, as indicated. The incorporation of ^35^S-methionine into proteins synthesised *de novo* was determined by SDS–PAGE and phosphorimaging (panel D).

To simplify certain plasmid constructions and to more closely mimic those constructs analysed in yeast [7], the [2A-PAC] portion of the pPDF18 polyprotein was deleted to create plasmid pPDF45ΔB, encoding [GT-EYFP-2A-ECFP] (Fig. 1A). Transfection of HeLa cells with pPDF45ΔB produced the same pattern of fluorescence as that obtained with pPDF18: yellow fluorescence in the Golgi and cyan fluorescence in the ER (Fig. 1B). When the GT type II signal-anchor in pPDF45ΔB was replaced by a type I signal sequence from the ER luminal protein calreticulin, both fluorescence signals were located in the ER (data not shown).

We suspected that the difference between the observations made in yeast and mammalian cells could lie either in; (i) some peculiarity of the signal sequences used in these analyses or (ii) the nature of the sequences upstream of 2A. To address the first possibility we replaced the GT Golgi targeting sequence with either of the two signal sequences used in the yeast experiments (αF and Dap2p – D_N_αF), obtaining plasmids pPDF90 (encoding [D_N_αF-EYFP-2A-ECFP]) and pPDF91 (encoding [αF-EYFP-2A-ECFP]). The yeast αF signal sequence has been shown to function within mammalian cells [30, 31]. Transfection of HeLa cells with these plasmids resulted in a similar pattern of fluorescence: both yellow and cyan signals in the ER. Again, it appeared that in both cases ECFP apparently ‘slipstream’ translocated into the ER (Fig. 1B). This effect was not, therefore, due to the nature of the signal sequences.

**Figure 2:**
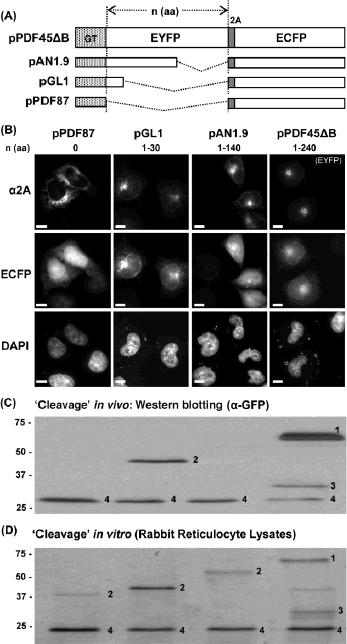
Effect of deletion of sequences upstream of 2A. EYPF sequences upstream of 2A were deleted (panel A). The number of residues between the end of the transmembrane domain of the GT signal sequence and the N-terminal residue of 2A are shown above the appropriate images in panel B. HeLa cells were transfected with plasmid constructs and images taken 24 h later (scale bar = 10μm).The localisation of EYFP and ECFP was determined by their native fluorescence, whilst the localisation of the deletion forms of EYFP was determined using anti-2A antibodies (panel B). Cell lysates were prepared, separated by SDS–PAGE and subsequently Western blotted using anti-GFP antibodies (panel C). *In vitro* translation systems programmed with constructs as indicated and the incorporation of ^35^S-methionine determined by SDS–PAGE and phosphorimaging. The translation products are denoted as; 1 = [GT-EYFP-2A-ECFP], 2 = [GT-ΔEYFP-2A-ECFP], 3 = [GT-EYFP-2A] and 4 = [ECFP] (panel D). *Note:* the [GT-ΔEYFP-2A] products from pAN1.9/pGL1 and the [GT-2A] product from pPDF87 were too small to be resolved in this gel system. Lanes in PAGE analyses of proteins (panels C and D) were derived from constructs used to produce the cell images shown in panel B, above.

**Figure 3:**
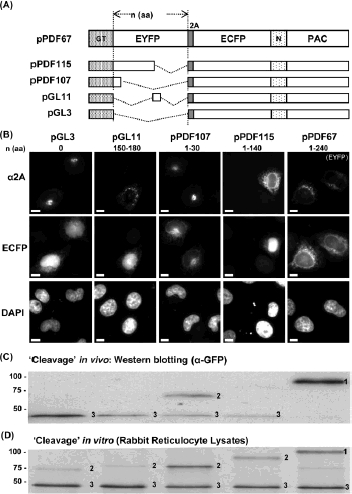
Effect of inclusion of a nuclear localisation signal. To visualise the partitioning of ECFP between the cytoplasm and the ER, we included a nuclear localisation signal (N; together with PAC) such that any cytoplas-mic [ECFP-N-PAC] would be localised in the nucleus (panel A). The number of residues between the end of the transmembrane domain of the GT signal sequence and the N-terminal residue of 2A are shown above the appropriate images in panel B. HeLa cells were transfected with plasmid constructs and images taken 24 h later (scale bar = 10 μm). The localisation of EYFP and ECFP was determined by their native fluorescence, whilst the localisation of the deletion forms of EYFP was determined using anti-2A antibodies (panel B). Cell lysates were prepared, separated by SDS–PAGE and subsequently Western blotted using anti-GFP antibodies (panel C). Rabbit reticulocyte *in vitro* translation systems were programmed with constructs as indicated and the incorporation of ^35^S-methionine determined by SDS–PAGE and phosphorimaging. Translation products are denoted as; 1 = [GT-EYFP-2A-ECFP-N-PAC], 2 = [GT-ΔEYFP-2A-ECFP-N-PAC] and 3 = [ECFP-N-PAC]. Note: the gel system we used was designed to resolve the [GT-ΔEYFP-2A-ECFP-N-PAC]/[GT-EYFP-2A-ECFP-N-PAC] products from [ECFP-N-PAC], and the [GT-ΔEYFP-2A] products from pPDF115/pPDF107/pGL1.The[GT-2A] product from pGL3 were not resolved in this system. Lanes in PAGE analyses of proteins (panels C and D) were derived from constructs used to produce the cell images shown in panel B, above.

To address the second possibility we analysed a panel of deletions within EYFP In this, and in all subsequent cases below, the numerals given in plasmid designations indicates the residues present in the EYFP deletion forms. The 2A sequence used in these analyses was derived from plasmid pSTA1/34 [6]. Following the 2A reaction the upstream protein bears, therefore, a C-terminal (2A) extension of 23aa. Initially we deleted all EYFP sequences, creating plasmid pPDF87 (encodes [GT-2A-ECFP]; Fig. 2A).We followed the fate of the [GT-2A] protein using an anti-2A mAb (kind gift of Dr. Dario A. Vignali). Whilst this antibody recognises 2A C-terminal extension of proteins, it does not recognise 2A within uncleaved fusion proteins (Fig. 1C). Transfection of HeLa cells with plasmid pPDF87 resulted in [GT-2A] localising to the Golgi and ER, with ECFP diffused throughout the cytoplasm but, inexplicably, the vast majority localising to the nucleus (Fig. 2B). Whilst the protein upstream of 2A ([GT-2A]) had entered the exocytic pathway as expected, the protein downstream had not. We wished to confirm ECFP had not entered the exocytic pathway by intensifying the fluorescence signal from ECFP by targeting the protein to the nucleus. A modified construct, pGL3, encoding [GT-2A-ECFP-N-PAC] (Fig. 3A) was expressed in HeLa cells. As before, [GT-2A] localised to the Golgi, whilst [ECFP-N-PAC] predominantly localised to the nucleus. In addition, a clear, but weak, signal could now be observed within the ER (Fig. 3B).

To further alter the sequence context immediately upstream of 2A, a range of deletions were made in the C-terminal region of EYFP. Plasmids pGL1 and pPDF107 both encode the same small fragment of the N-terminus of EYFP between the GT signal and the 2A. When either construct was transfected in HeLa cells, the ECFP (or, in the case of pPDF107 [ECFP-N-PAC]) apparently ‘slip-streamed’ in the ER (as with pPDF45ΔB), while the [GT-ΔEYFP_1-30_-2A] localised to the Golgi (Figs. 2A, B and 3A, B). In both cases a substantial amount of the full-length translation product was observed – the 2A reaction was inhibited (see below). Interestingly, when this N-terminal region of EYFP was also deleted (pPDF87, Fig. 2A; pGL3, Fig. 3A), the 2A reaction was no longer inhibited (see below) and the vast majority of the protein downstream of 2A was not translocated into the ER (Figs. 2B, 3B).

**Figure 4:**
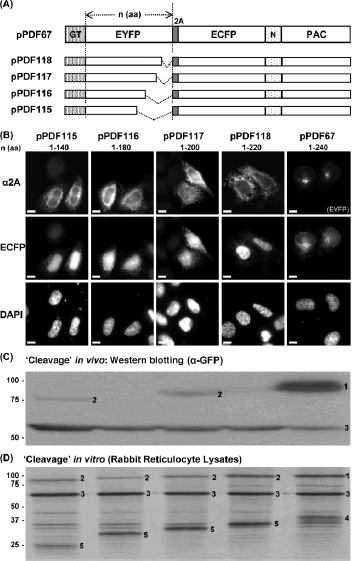
Effects of deletion of sequences immediately upstream of 2A. A panel of constructs were assembled with a nested set of deletions upstream of 2A. As described above, the ECFP bore a nuclear localisation signal (panel A). The number of residues between the end of the trans-membrane domain of the GT signal sequence and the N-terminal residue of 2A are shown above the appropriate images in panel B. Cell lysates were prepared, separated by SDS–PAGE and subsequently Western blotted using anti-GFP antibodies. Rabbit reticulocyte *in vitro* translation systems were programmed with constructs as indicated and the incorporation of^35^S-methionine determined by SDS–PAGE and phosphorimaging. Translation products are denoted as; 1 = [GT-EYFP-2A-ECFP-N-PAC], 2 = [GT-ΔEYFP-2A-ECFP-N-PAC], 3 = [ECFP-N-PAC], 4 = [GT-EYFP-2A] and 5 = [GT-ΔEYFP-2A]. Note: the higher molecular masses of the [GT-ΔEYFP-2A] products allowed complete resolution in this gel system. The ‘extra’ bands arise from internal initiation events in this batch of reticulocyte lysate. Lanes in PAGE analyses of proteins (panels C and D) were derived from constructs used to produce the cell images shown in panel B, above.

Plasmid pAN1.9 encodes EYFP with a much smaller deletion at the C-terminus ([GT-ΔEYFP_1-140_-2A-ECFP]; Fig. 2A). Here, the result obtained was similar to that using pPDF87: while the [GT-ΔEYFP_1-140_-2A] product upstream of 2A localised to the Golgi, the downstream product (ECFP) localised to the cytoplasm and nucleus (Fig. 2B). A similar plasmid, pPDF115, encodes [GT-ΔEYFP_1-140_-2A-ECFP-N-PAC] (Figs. 3 and 4A). Transfection with this plasmid showed the protein [GT-ΔEYFP_1-140_-2A] upstream of 2A had, again, entered the exocytic pathway. Although bearing a GT signal, this protein was distributed in both the ER and Golgi. The protein downstream of 2A (ΔECFP-N-PAC]) was localised in the nucleus, with a much weaker signal from the ER (Figs. 3 and 4B).

If the context immediately upstream of 2A is crucial in producing this apparent slipstream translocation, then small deletions in the C-terminal region of EYFP might be sufficient to reduce or eliminate this effect. A series of constructs were made with progressive deletions within EYFP (plasmids pPDF118, pPDF117, pPDF116 and pPDF115; Fig. 4A). In all cases, the protein upstream of 2A was translocated into the ER and the downstream [ECFP-N-PAC] localised to the nucleus (with a weak signal in the ER). The data from all of these plasmids contrasts with the ER localisation of [ECFP-N-PAC] in cases when the C-terminal region of EYFP was present immediately upstream of 2A (plasmid pPDF67; Figs. 3 and 4B). Deletion of just the C-terminal 20aa of EYFP (pPFD118) relieved the inhibition of the 2A reaction.

### 3.2 Effect of the upstream context on the 2A reaction in vitro and in vivo

We routinely monitor the cleavage activity of 2A-containing artificial polyproteins using coupled transcription/translation rabbit reticulocyte cell-free translation systems (Figs. 1–4, panel D). Using constructs encoding artificial polyproteins such as [EYFP-2A-ECFP] we had correlated the activities we had observed using *in vitro* translation systems with that observed within transfected cells (analysed by Western blotting). In both cases cleavage was high – indeed higher (almost 100%) *in vivo*. We took it, therefore, that the *in vitro* analyses were a good indication of cleavage *in vivo*.

When we investigated the cleavage *in vivo* of the polyproteins we had created that gave rise to the apparent slipstream sub-cellular localisation – those bearing N-terminal signal sequences – we found a very different picture. For example, in the case of pPDF45ΔB and pPDF67, translation *in vitro* produced only a small amount of the full-length translation product (Figs. 2–4, panel D), whilst expression *in vivo* showed this form to be the major product with the ‘cleaved’ forms being weak bands - sometimes visible only with prolonged exposure (Figs. 2–4, panel C). The *in vivo* analyses of cleavage showed a major difference in efficiency between those constructs which gave rise to the apparent ‘slipstream’ effect and those which did not. The slipstream effect showed complete correlation with low efficiency of the 2A reaction – evidenced by the levels of the full-length translation products (Figs. 1–4, panel D). Taken together these results strongly suggest that in the case of proteins targeted to the exocytic pathway the immediate upstream context of 2A may strongly influence the efficiency of the reaction. In the case of pGL1 and pPDF107 the same number of residues lie between the end of the transmembrane domain of the GT signal sequence and the N-terminal residue of 2A (Figs. 2A and 3A, respectively). We designed pGL11 ([GT-ΔEYFP_150-180_-2A-ECFP-N-PAC]) to encode the same number of residues between the GT signal and 2A as in pGL1/pPDF107, but in pGL11 the upstream context for 2A is identical to that in pPDF116 – a construct that showed a highly efficient 2A reaction (Fig. 4C). Upon transfection of cells with plasmid pGL11, the translation product upstream of 2A ([GT-ΔEYFP_150-180_-2A]) was detected in the Golgi (immunofluorescence using anti-2A antibodies) whilst the translation product downstream of 2A ([ECFP-N-PAC]) was localised predominantly in the nucleus. The 2A reaction was highly efficient (using *in vitro* translation systems and *in vivo*; Figs. 3C, D). These data are consistent with the notion that, in the case of proteins entering the exocytic pathway, the context immediately upstream of 2A may strongly inhibit the 2A reaction.

## 4 Discussion

2A and 2A-like sequences are now widely used as a tool for co-expression in biomedicine and biotechnology due to (i) their shortness (20–30aa), (ii) highly efficient cleavage, (iii) the uniform stoi-chiometry of the cleavage products and (iv) their activity in all eukaryotic cell-types tested to date. Many laboratories have successfully used 2As to co-express proteins targeted to the exocytic pathway. For example, the p35 and p40 chains of interleukin-12 (IL-12) were cleaved highly efficiently.

Both chains bore their native signal sequences and active IL-12 was secreted into the media [32]. Similarly, the heavy and light chains of antibodies cleaved highly efficiently, were assembled and secreted [33]. The α- and β-subunits of the T-cell receptor (in both orientations) cleaved highly efficiently, assembled and were localised to the plasma membrane [13]. Human iduronidase alpha-L (IDUA) was targeted to the secretory pathway whilst a marker protein downstream of 2A *(Discosoma* spred fluorescent protein, DsRed2) localised to the cytosol [34].

In our previous study with fluorescent polyproteins where the first protein was targeted to the secretory pathway and the protein downstream of 2A was cytosolic, we attributed the unexpected ER sub-cellular distribution of the downstream protein to slipstream translocation [28]. Here we show a complete correlation between those polyproteins displaying the apparent slipstream effect and those with low levels of the 2A reaction *in vivo* – monitored by Western blotting. A large proportion of the translation products are uncleaved, leading to translocation of the fusion protein into the exocytic pathway.

Analyses of protein targeting using a control construct (pPDF93), encoding [GT-EYFP-ECFP], showed that (i) the fusion protein partitions between the Golgi and ER (mainly ER) and (ii) both proteins fluoresce in both compartments – producing complete co-localisation upon merging the images [28]. However, when 2A is present between the fluorescent proteins (pPDF45ΔB), whilst the fusion protein partitions between the Golgi and ER, the [GT-EYFP-2A] product formed by the 2A reaction (strongly fluorescent) localises to the Golgi, whilst we assume the [ECFP] product (much more weakly fluorescent than EYFP) is diffused throughout the cytoplasm and nucleus and not detectable.

In the case of constructs with N-terminal signal sequences, the source of the inhibitory effect of the EYFP C-terminal sequences (immediately upstream of 2A) must lie in the interaction with the translocon complex, since they cleave highly efficiently using *in vitro* translation systems. For example, the [GT-EYFP-2A-ECFP-N-PAC] polyprotein encoded by pPDF67 shows high level of cleavage *in vitro*, while *in vivo* most of the protein detected by anti-GFP antibodies was in the full-length, uncleaved, form [GT-EYFP-2A-ECFP-N-PAC], with very little detected as the cleavage products [GT-EYFP-2A] and [ECFP-N-PAC] (Figs. 3, 4 panels C,D).

We have proposed that nascent 2A forms an α-helix with a tight-turn at its C-terminus. The helical portion is proposed to interact with the ribosome exit tunnel such that the C-terminal portion is sterically constrained within the peptidyl-transferase centre of the ribosome [4, 5]. The ester linkage between 2A and transfer RNA (tRNA)^gly^ is precluded from nucleophilic attack by the prolyl-tRNA in the A site, effectively ‘jamming’ translation. We have recently shown that this block is relieved by the action of translation release factors 1 and 3 [9].

**Figure 5:**
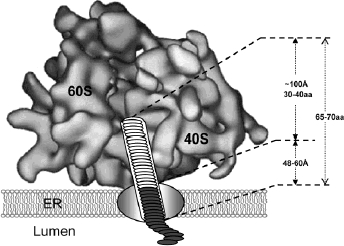
Position of 2A and sequences immediately upstream in the ribosome:translocon complex. In the case of polyproteins bearing an N-terminal signal sequence, the 2A oligopeptide (white ovals) and the sequences immediately upstream (grey ovals) are predicted to be located within the ribosome exit tunnel and translocon pore, respectively. The lengths ofthe exit tunnel and translocon pore, plus the lengths of pep-tides (amino acids) which may be accommodated [44–49] are also shown.

Residues that influence the 2A-mediated cleavage of cytosolic proteins map to within 30aa of the cleavage site. This is consistent with many pro-teinase protection studies which have shown the length of nascent peptide within the ribosome exit tunnel to be 30–40aa (Fig. 5; reviewed in [35]). The eukaryotic ribosome exit tunnel is ∼ 100 Å long and has an average diameter of ∼20 Å [36].

The C-terminal 20aa of EYFP upstream of 2A that inhibited the 2A reaction are 28–48aa distal from the peptidyl-transferase centre – since we know that the C-terminus of 2A (23aa long + 4aa linker) is in the P site when cleavage occurs (Fig. 5). Disulphide bond formation, photochemical cross-linking, proteinase protection, glycosylation acceptor site and protein folding studies performed upon nascent proteins transiting into the lumen of the ER show that 65–70aa are present within the ribo-some:translocon complex [37–42] (Fig. 5).The consensus arising from structural studies on Sec61 or Sec61/ribosome complexes is that the protein conducting channel is formed at the interface of an oligomeric assembly – probably a tetramer – of the Sec61α,γ,β complex. The channel is co-axial with the ribosome exit tunnel and is thought to be some 48-60 Å long [43–48].

Theoretical work suggests that nascent proteins adopt an α-helical conformation [49] stabilised by the exit tunnel [50]: indeed, dynamic simulations suggested 2A adopted such a conformation [4]. Taken together, these data suggest that at the stage in elongation when cleavage occurs (the C-terminal glycyl-tRNA^gly^ of 2A in the P site of the ribosome peptidyl-transferase centre), the C-terminal 20aa of the protein upstream (EYFP) most probably lie within a region defined by the interface between the ribosome and Sec61 complex and the translocon tunnel itself (Fig. 5). Interactions between the nascent protein and the translocon tunnel may affect the conformation of 2A within the ribosome exit tunnel and, in consequence, the C-terminal tight-turn of 2A in the peptidyl-transferase centre itself - inhibiting the 2A reaction.

The problem we have identified in the co-expression of certain proteins targeted to the exocytic pathway has significance for the biotechnological utilities of 2A. The LOCATE sub-cellular localisation database indicates that of all human proteins currently analysed, ∼40% are either secreted from the cell, located within the lumen/membranes of cytoplasmic vesicular structures (excluding mitochondria), or are plasma membrane proteins (http://locate.imb.uq.edu.au/). Given that such a high proportion of cellular proteins are initially translocated into the ER, the ability to co-express multiple proteins targeted to such sites is essential.

In this study we identified two regions of EYFP which, *when placed immediately upstream of2A*, inhibited the 2A reaction; (i) the C-terminal region (residues 220–240: pPDF118) and (ii) the N-terminal region (residues 1–29: pGL1/pPDF107). However, in constructs we assembled designed to co-express influenza haemagglutinin (HA) or neu-raminidase (NA) linked *via* 2A to fluorescent proteins ([HA-2A-CherryFP] or [NA-2A-CherryFP]), we observed the same effects we describe here: very high-level cleavage using translation systems *in vitro*, whilst expression *in vivo* showed the fluorescent proteins localised in the ER and not to the cytoplasm – again, the apparent slipstream effect.

This inhibition of the 2A reaction may be overcome in two ways. Firstly, by the use of longer versions of 2A which incorporate a 39aa tract from the C-terminus of protein 1D, immediately upstream of 2A in the FMDV polyprotein [6]. This extension does not interact with the translocon pore to affect the activity of 2A in the ribosome – effectively ‘insulating’ the 2A sequence from the upstream protein. A number of studies suggest that cleavage efficiency may be improved by using a flexible Gly–Ser–Gly or Ser–Gly–Ser–Gly linker sequence separating the upstream protein from the 2A sequence [51–53]. Recent work by Yang *et al.* (2008) combined a furin cleavage site and a V5 oligopep-tide spacer sequence immediately upstream of 2A [54]. Furin is a proteinase enriched in the Golgi, with a canonical recognition site of -R-X-↓(R/K)-R-. For proteins targeted to, or transiting through, the Golgi, incorporation of this site results in proteoly-sis. This strategy may provide an excellent solution since it removes the extended 2A linker [55]. We have used a linker comprising the furin proteinase cleavage site, 39aa of 1D, plus 2A (derived from plasmid pSTA1/31; [6]) to overcome this problem. In our influenza HA/NA co-expression studies the use of this linker resulted in the correct localisation of the cherry fluorescent protein to the cytoplasm, and not the exocytic pathway (S. Vater, pers.comm.).

The second solution lies in the basic design – the “gene” order – of the polyprotein. Previously described 2A-based multigene vectors have revealed the 2A region functions properly within different contexts, but the cleavage efficiency varies as flanking context changes. By swapping the order of proteins in several artificial polyproteins the stoichiometry was affected by the gene upstream of 2A [56, 57]. If a problematic secreted/membrane protein is identified, then this could be incorporated as the C-terminal component of the polyprotein system. It should be noted in this context that in mammalian and yeast cells an N-terminal proline (such as that produced by 2A-mediated cleavage) confers a long half-life (>20 h) on proteins (http://www.expasy.ch/tools/protparam-doc.html).

## Abbreviations:

CMV: cytomegalovirus
ECFP: enhanced cyan fluorescent protein
ER: endoplasmic reticulum
eRF1: eukaryotic release factor 1
eRF3: eukaryotic release factor 3
EYFP: enhanced yellow fluorescent protein
GFP: green fluorescent protein
GT: β-1,4 galactosyltransferase
HA: haemagglutinin
NA: neuraminidase
PAC: puromycin resistance
tRNA: transfer RNA
